# The influence of parental educational expectations on primary school students’ social-emotional competence: the chain mediation effect of peer relationships and academic self-efficacy

**DOI:** 10.3389/fpsyg.2025.1683211

**Published:** 2025-10-22

**Authors:** Lianghong Yang, Kaizhou Chen, Suling Ma, Yan Lei

**Affiliations:** Faculty of Teacher Education, Lishui University, Lishui, China

**Keywords:** primary school students, parental educational expectations, social-emotional competence, peer relationships, academic self-efficacy, chain mediation effect

## Abstract

**Purpose:**

In the context of deepening educational reform and emphasizing comprehensive development, social-emotional competence is crucial to the growth of primary school students. Parental educational expectations are a core element of the family micro-system, but their influence mechanism has yet to be explored in depth. Based on the ecosystem theory, this study examines the influence of parental educational expectations on primary school students’ social-emotional competence, as well as the chain mediation effect of peer relationships and academic self-efficacy.

**Methods:**

A questionnaire survey was conducted on 1,653 primary school students in grades 3–5 in Zhejiang Province using an adapted parental educational expectations questionnaire, a primary school students’ social-emotional competence questionnaire, a peer relationships questionnaire, and an academic self-efficacy questionnaire. AMOS 28.0 was used to construct and test a chain mediation effect model.

**Results:**

The analysis found that parental educational expectations could directly and positively predict primary school students’ social-emotional competence (*β* = 0.22, *p* = 0.001, accounting for 28.0%); peer relationships partially mediated the relationship between parental educational expectations and social-emotional competence (*β* = 0.26, *p* < 0.001, accounting for 34.0%); academic self-efficacy partially mediates the relationship between parental educational expectations and social-emotional competence (*β* = 0.19, *p* < 0.001, accounting for 24.1%); parental educational expectations indirectly affect social-emotional competence through the chain mediation effect of “peer relationships → academic self-efficacy” (*β* = 0.11, *p* < 0.001, accounting for 13.9%).

**Conclusion:**

Parental educational expectations not only directly promote primary school students’ social-emotional competence, but also have an indirect influence through peer relationships, the independent mediating effect of academic self-efficacy, and the chain mediation effect of the two. This study reveals the mechanism of the synergistic effects of family, peers, and individual psychological resources, providing inspiration for educational practice.

## 1 Introduction

Social-emotional competence is a crucial factor in promoting students’ holistic development (Wang et al., 2021), and they play a pivotal role in an individual’s career success and overall life satisfaction (Yuan, 2021). The United States, European Union countries, Japan, Singapore, and other countries have increasingly emphasized the cultivation of social-emotional competence in adolescents aged 10–15, positioning it as a core competence and educational strategy priority for talent in the 21st century (Qu and Liu, 2020). In China, strengthening the social-emotional competence learning of primary and secondary school students has become an inevitable requirement for the transformation and upgrading of basic education in the new era (Mao and Du, 2021). However, the cultivation of adolescents’ social-emotional competence is a difficult task in the comprehensive deepening reform of the education system. Chinese families and schools have not done enough to cultivate students’ social-emotional competence (Li, 2023; Yu and Jiang, 2017), and students face many challenges in their social-emotional development and interpersonal relationships (Ding et al., 2022).

Bronfenbrenner’s ecological theory emphasizes that individual development is embedded in multiple environmental systems, and parental educational expectations, as a key factor in the micro-system of the family environment, play an important role in promoting individual development (Li et al., 2021). Research shows that parental educational expectations are significantly related to students’ academic performance and cognitive abilities (Yang and Wang, 2025; Zhuang et al., 2024), and they can affect students’ learning engagement and psychological stress (Zhang et al., 2020; Ma and Zhang, 2022). Although there has been a wealth of research in this field, there is still insufficient discussion of the mechanism of influence of parental educational expectations on primary school students’ social-emotional competence.

Although related studies have focused on the mediating role of peer relationships and academic self-efficacy, such as the mediating role of peer relationships between social support and proactive health behaviors in adolescents (Lu et al., 2024) the mediating role of academic self-efficacy between college students’ mental health and academic performance (Song and Hu, 2024). This shows the important mediating role of peer relationships and academic self-efficacy in individual mental health and emotional development, but few studies have considered both in the same model. Furthermore, from the perspective of the ecosystem theory, whether the two can mediate between parental educational expectations and social-emotional competence remains to be explored.

Therefore, this study aims to explore the impact and mechanism of parental educational expectations on primary school students’ social-emotional competence, and attempts to analyze its specific impact path and effect through the chain mediation effect of peer relationships and academic self-efficacy.

## 2 Literature review and research hypothesis

### 2.1 Parental educational expectations and social-emotional competence

Parental educational expectations refer to parents’ realistic expectations and beliefs about their children’s future academic achievements, covering their expectations regarding the type of education their children receive, the nature of the school, academic achievements, and home-school cooperation. They reflect parents’ judgments and aspirations regarding their children’s ultimate academic qualifications or educational level (Yamamoto and Holloway, 2010; Luo, 2015; Guo and Luo, 2019). In terms of specific dimensions, some scholars divide them into five dimensions: academic performance, future development, interpersonal relationships, behavioral performance, and physical and mental qualities (Cheng, 2010). Other studies divide parental educational expectations into four dimensions from the perspective of career development expectations: autonomous development, achievement status, safety and comfort, and professional conformity (Hou et al., 2012). These dimensions not only encompass parents’ direct expectations for students’ educational achievements, but also involve expectations for students’ skill development, career planning, and personal growth. Based on the above research, it can be seen that parental educational expectations are a comprehensive concept that includes parents’ expectations for students’ future development and physical and mental qualities. Therefore, this study defines it as parents’ attitudes and judgments about students’ future development and physical and mental qualities, including expectations for students’ future development and physical and mental health.

Social-emotional competence is an important part of non-cognitive abilities (Organisation for Economic Co-operation and Development, 2015). It is a key skill for students during their school years and future social life (Yang et al., 2019), and has a significant promotional effect on students’ ability to cope with setbacks and academic performance (Ashdown and Bernard, 2012). Good social-emotional competence can enhance students’ sense of belonging to school, reduce the occurrence of problem behaviors, and improve students’ mental health (Sklad et al., 2012; Korpershoek et al., 2016; Qu and Chen, 2023). Its core components include an individual’s ability to recognize, identify, regulate, and effectively express emotions, representing the essence of social interaction skills (Denham, 2006). It not only involves how individuals understand and regulate their own thoughts, emotions, and behaviors but also emphasizes the establishment of positive interpersonal networks and making appropriate decisions to address challenges in social life (Song and Kim, 2022), exhibiting characteristics such as complexity, contextualization, practicality, and developmental nature (Mao and Yu, 2024). Therefore, this study believes that social-emotional competence encompasses an individual’s emotional cognition, regulation, and effective expression, involving an individual’s understanding and regulation of their own thoughts, emotions, and behaviors, as well as their ability to establish positive interpersonal relationships and assume collective responsibility in social interactions. Ultimately, it aims to enable individuals to actively respond to challenges and adapt to society.

In terms of dimensionality, different scholars and research institutions have proposed a variety of models of social-emotional competence. For example, the five-dimensional model proposed by the Collaborative for Academic, Social and Emotional Learning emphasizes the importance of self-awareness, self-management, social awareness, interpersonal skills, and responsible decision-making (Durlak et al., 2011). This model comprehensively covers the core elements of social-emotional competence and provides a theoretical framework for subsequent empirical research. The “Social and Emotional Learning” project initiated by the Chinese Ministry of Education and the United Nations Children’s Fund combines China’s actual circumstances and proposes a six-dimensional model encompassing self-awareness, self-management, others’ awareness, others’ management, collective awareness, and collective management, providing an important reference for promoting the development of social and emotional education in China (Chen and Mao, 2016). Based on the above research, this study divides social-emotional competence into three dimensions: self-awareness, interpersonal relationships, and collective responsibility, in order to further focus on and deepen social-emotional competence in the context of Chinese education.

In the study of the relationships between parents and students’ social-emotional competence, some scholars have used the “Big Five Personality” as an assessment framework and found, based on the SESS2019 dataset, that parental educational expectations have an impact on the social-emotional competence of middle school students in China, South Korea, the United States, and Finland (Niu, 2024). Focusing on Chinese students, primary school students and secondary school participants in the assessment demonstrated significant differences in social-emotional competence across educational stages. Specifically, primary school students excelled in multiple competencies, ranking among the top globally in task competence and collaboration skills. In contrast, secondary students generally performed at the international mid-range level. While their collaboration skills were relatively strong, they showed notable deficiencies in dimensions such as curiosity and vitality (Tang, 2025). This disparity indicates uneven development of social-emotional competence across different educational stages. Considering that primary school students are in the early stages of psychological and cognitive development, they are highly dependent on their parents, highly compliant, and have relatively limited independent judgment abilities. This group may be more likely to internalize and comply with their parents’ educational expectations. Therefore, this study will focus on primary school students, aiming to explore the specific impact of parental educational expectations on the social-emotional competence of this younger age group. Based on this, this study proposes hypothesis, H1:

*H1:* Parental educational expectations have a positive impact on primary school students’ social-emotional competence.

### 2.2 The mediating role of peer relationships

Peer relationships refer to interpersonal relationships formed between people of similar age and psychological development through interaction (Zou, 1998). As one of the micro-systems in the ecological theory perspective, peer relationships can influence individual development (Bronfenbrenner, 1977), and provide companionship, emotional support, and entertainment opportunities for students’ growth (Oberle et al., 2018).

Research shows that families play an important role in the formation and development of students’ peer relationships. Studies have pointed out that parental educational expectations are significantly positively correlated with peer relationships (Chen, 2020), which can affect the position of junior high school students in peer relationships (Yang and Zhu, 2013). However, the relationship between parental educational expectations and peer relationships is not always linear, and its dynamics may be complex. For instance, when there is a significant gap between parental educational expectations and students’ self-held educational expectations, it can affect the quality, size, and structure of students’ peer relationships (Rutherford, 2015). Further research indicates that the gap between parental and student educational expectations plays a moderating role in the relationship between these expectations and both academic achievement and peer relationship quality (Zhao and Zhao, 2022). This suggests that parental educational expectations are closely linked to students’ peer relationships.

In addition, peer relationships have an important impact on the development of students’ social-emotional competence. Close peer relationships can influence the development of students’ behavioral regulation and social attitudes, suggesting that high-quality peer relationships are an important way to cultivate social-emotional competence (Heinze et al., 2018). Peer relationships are often divided into two dimensions: peer acceptance and peer rejection. Peer acceptance is significantly positively correlated with social-emotional competence (Yang et al., 2017), and helps to reduce the impact of negative emotions, prompting individuals to regulate their emotions and inhibit impulses in adaptive ways, thereby developing social-emotional competence (Arató et al., 2022). Based on the above research, this paper proposes hypothesis, H2:

*H2:* Peer relationships play a mediating role between parental educational expectations and social-emotional competence.

### 2.3 The mediating role of academic self-efficacy

Bandura defines self-efficacy as an individual’s assessment of their ability to organize and execute the actions necessary to achieve a specific performance (Bandura, 1986). In the field of learning, this concept is derived from academic self-efficacy, which refers to learners’ belief in their ability to successfully achieve educational goals (Elias and MacDonald, 2007). Based on Bandura’s views, Chinese scholar Liang (2000) divided academic self-efficacy into two dimensions: learning ability efficacy and learning behavior efficacy, and compiled a relevant questionnaire that is widely used in Chinese psychological research. On this basis, this study divides academic self-efficacy into three dimensions: academic confidence, knowledge application, and learning approaches. Among them, academic confidence reflects an individual’s overall confidence in their own academic ability; knowledge application emphasizes an individual’s ability to apply what they have learned to real-life situations; and learning approaches focuses on an individual’s mastery and application of learning strategies and methods.

Research shows that academic self-efficacy plays an important role in the development of students’ social and emotional competence. The Tuck Family Foundation and the Children’s Trends organization believe that academic self-efficacy is conducive to the formation and improvement of students’ social and emotional skills (Collaborative for Academic, Social, and Emotional Learning (CASEL), 2015), indicating that students’ confidence in their own learning abilities may be an important factor in the development of good social-emotional competence. Some scholars have pointed out that academic self-efficacy is significantly related to the positive emotions of junior high school students (Tang et al., 2022), further confirming the positive impact of academic self-efficacy on an individual’s emotional development, and that social-emotional competence, as an important part of an individual’s emotions, may also be affected by it.

From the perspective of the family environment, parental educational expectations, as an important socialization factor, have a significant impact on students’ academic self-efficacy (Tang et al., 2022; Guo et al., 2019). When parental educational expectations are consistent with students’ self-educational expectations, students’ academic self-efficacy will be enhanced; conversely, students’ academic self-efficacy may be negatively affected (Tang et al., 2022; Guo et al., 2019). Based on the above research, this paper proposes hypothesis, H3:

*H3:* Academic self-efficacy mediates the relationship between parental educational expectations and social-emotional competence.

### 2.4 The chain mediation effect of peer relationships and academic self-efficacy

According to social cognitive theory, the formation and development of self-efficacy are rooted in specific social interaction contexts, and the feelings and evaluations individuals gain through interpersonal interactions are important sources of self-efficacy (Bandura, 1986). Research has shown that self-efficacy is influenced by peer interactions (Ruegg, 2018; Sökmen, 2021; Shyr et al., 2021). Specifically, peer interaction support is crucial for students to develop positive attitudes, enhance self-confidence, and improve their ability to make learning judgments (Chu and Chu, 2010). Peer modeling facilitates adolescents’ cognitive, emotional, and behavioral development, as adolescents are influenced by role models in peer modeling, which promotes the development of their self-efficacy (Lee et al., 2021). In addition, peer cooperation has an impact on self-efficacy (Lee and Evans, 2019). Peer cooperation is based on good peer relationships, which suggests that peer relationships may be an important factor in promoting the development of academic self-efficacy. At the same time, research indicates that individuals with higher academic self-efficacy often exhibit greater self-confidence. This confidence manifests not only in academic settings but may also extend to social interactions, thereby contributing to the development of more positive peer relationships (Huang, 2015). In the ecosystem theory, the family and peers are two microsystems whose synergistic effects can promote the healthy development of students. Parental educational expectations, as a micro-system element of the family, can influence peer relationships, which in turn can influence academic self-efficacy, which in turn influences primary school students’ social-emotional competence. Therefore, this paper proposes, H4:

*H4:* Peer relationships and academic self-efficacy play a chain mediation effect between parental educational expectations and primary school students’ social-emotional competence.

**Figure 1:**
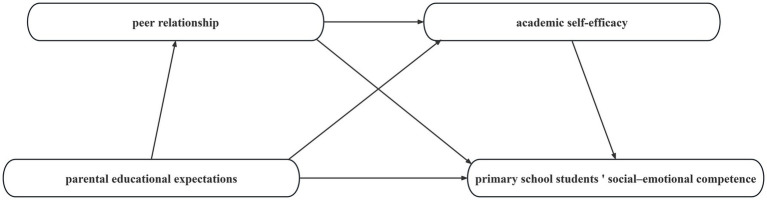
Theoretical model diagram of this study.

In summary, the theoretical model constructed in this study is shown in Figure 1.

## 3 Methods

### 3.1 Participants

China is currently undergoing a critical period of deepening educational reform, emphasizing quality education and the comprehensive development of students. Beyond the transmission of knowledge, there is a greater focus on the multidimensional growth of students’ emotional attitudes, values, and social skills. This study was conducted in Zhejiang Province, China, which has a solid economic foundation and abundant educational resources. It has achieved remarkable results in educational innovation, balanced development, and the implementation of quality education, and is at the forefront of educational reform. It is one of the strongest provinces in terms of education and provides a good environment for researching the cultivation of primary school students’ social-emotional competence. This study collected data offline through paper questionnaires. This study selected third, fourth, and fifth-grade primary school students as the survey subjects. To ensure research quality and data reliability, the questionnaire survey was conducted through team collaboration. Prior to formal administration, the research team organized standardized training sessions covering questionnaire content, administration procedures, and key considerations. During the survey implementation, the research team received substantial support and cooperation from local education authorities. Specifically, two trained researchers entered each classroom to distribute questionnaires, first providing a unified explanation of the questions and completion requirements. If students encountered questions during the process, team members offered on-the-spot clarification to ensure consistent understanding of the questionnaire content among all participants, thereby safeguarding data validity and reliability. The questionnaire was anonymous to protect privacy, and all participants voluntarily participated on the basis of informed consent. Ultimately, researchers successfully collected 1,663 paper questionnaires, which were entered into China’s professional questionnaire collection platform “WJX” to export raw data. After review and correction using SPSS 27.0, the study obtained 1,653 valid data points. Among these, 50.8% were male and 49.2% were female; third-grade students accounted for 31.2%, fourth-grade students for 41.3%, and fifth-grade students for 27.5%.

### 3.2 Research tools

This study utilized four measurement tools. Among these, the Parental Educational Expectations Questionnaire, Peer Relationships Questionnaire, and Academic Self-Efficacy Questionnaire were adapted from the works of three researchers (Cheng, 2010; Guo and Zhang, 2003; Liang, 2000), while the Primary School Students’ social-emotional Competence Questionnaire was adapted from the Student social-emotional Competence Questionnaire compiled by the Chinese Ministry of Education-United Nations Children’s Fund “social-emotional Learning” Project Team. These questionnaires all use a five-point Likert scale (1 = completely disagree to 5 = completely agree), and Cronbach’s *α* and KMO values have been tested. Their validity and various fit indices are good.

#### 3.2.1 Parental educational expectations questionnaire

The Parental Educational Expectations Questionnaire was adapted from Cheng’s (2010) Parental Expectations Questionnaire. The original questionnaire demonstrated good reliability with a Cronbach’s α coefficient of 0.855. Given the low reliability of three items (Cronbach’s *α* = 0.649) after administration, this study removed these three items based on reliability analysis results to enhance the internal consistency and efficiency of the measurement tool, ultimately retaining six items. The questionnaire was divided into two dimensions: Future Development and Physical and Mental Health, with three items in each dimension. The Future Development dimension refers to parents’ expectations regarding students’ academic achievement and social status; while the physical and mental health dimension encompasses parental concerns and expectations regarding children’s physical health, lifestyle habits, and regular health checkups. The adjusted questionnaire yielded a Cronbach’s *α* coefficient of 0.701 and a KMO value of 0.714, indicating satisfactory internal consistency.

#### 3.2.2 Primary school students’ social-emotional competence questionnaire

The Social-Emotional Competence Questionnaire for Primary School Students used in this study was adapted from the Student Social-Emotional Competence Questionnaire developed by the Chinese Ministry of Education-UNICEF “Social-Emotional Learning” Project Group. The original questionnaire comprised six dimensions: self-awareness, self-management, perception of others, management of others, perception of the group, and management of the group, totaling 30 items. The revised questionnaire retains the original theoretical framework progressing from the individual to others and the collective. It consolidates the six dimensions into three: Self-Awareness, Interpersonal Relationships, and Collective Responsibility, each comprising three items. Self-Awareness assesses students’ recognition of their strengths, desire for respect, and ability to learn from successful experiences; Interpersonal Relationships measures students’ ability to understand differing opinions, initiate social interactions, and maintain harmonious relationships with others; Collective Responsibility reflects students’ sense of belonging to a group, willingness to maintain public environments, and sense of responsibility to take the lead in doing good deeds. Given that the research subjects are third- to fifth-grade elementary students, whose cognitive comprehension and sustained attention during testing are still developing, overly detailed dimension divisions and excessively long items may cause fatigue effects, impacting data quality. The integrated dimensions retain the core structure of the original questionnaire while better aligning with the cognitive and comprehension levels of upper primary school students, facilitating administration and expression. Results indicate that the adapted questionnaire achieved a Cronbach’s alpha coefficient of 0.846 and a KMO test coefficient of 0.881, demonstrating strong reliability and validity.

#### 3.2.3 Peer relationships questionnaire

The Peer Relationship Questionnaire is adapted from Professor Guo and Zhang’s (2003) Children and Adolescents Peer Relationship Scale. This questionnaire assesses children and adolescents’ perceptions of their peer relationships, with established reliability confirmed by prior research (Song, 2017). Through confirmatory factor analysis, this study identified three dimensions: Peer Acceptance, School Attitude, and Peer Friendship, each comprising four items. Peer Acceptance assesses students’ anxiety and concerns regarding their level of recognition and likability within peer groups; School Attitude measures students’ willingness, preference, and emotional experiences toward attending school; Peer Friendship reflects students’ perceived number of friends, quality of interactions, and level of peer support. Data analysis indicates that the adapted questionnaire demonstrates good reliability and validity, with a Cronbach’s *α* coefficient of 0.768 and a KMO value of 0.808.

#### 3.2.4 Academic self-efficacy questionnaire

The Academic Self-Efficacy Questionnaire, adapted from Liang Yusong’s revised Academic Self-Efficacy Scale, consists of 9 items. To align with the assessment needs of primary school students, the questionnaire is divided into three dimensions: Academic Confidence, Knowledge Application, and Learning Approaches, with three items per dimension. Academic confidence measures students’ self-assurance in their learning abilities and capacity to achieve good grades; knowledge application assesses students’ ability to integrate learned knowledge and apply it to solve real-world problems; learning approaches reflects the strategies and methods students employ to enhance learning outcomes. The questionnaire demonstrated a Cronbach’s α coefficient of 0.865 and a KMO value of 0.902, indicating strong reliability and validity, making it suitable for assessing academic self-efficacy among primary school students.

### 3.3 Methods of analysis

After entering the paper questionnaire data into the “WJX” platform, 1,653 valid questionnaires were subjected to missing value imputation and coding. SPSS 27.0 was used to calculate means, standard deviations, and percentages, and to perform descriptive statistical analyses. Normality tests were conducted on all variables based on skewness values, and the results met the requirements. In addition, this study used Amos 28.0 to construct a structural equation model to examine the effects of parental educational expectations, peer relationships, and academic self-efficacy on primary school students’ social-emotional competence, and to examine the chain mediation effect of peer relationships and academic self-efficacy between parental educational expectations and primary school students’ social-emotional competence.

## 4 Results

### 4.1 Common method bias test

To prevent common method bias, this study used Harman’s one-way analysis of variance method to calculate and test all items. The results showed that there were eight factors with eigenvalues greater than 1. The first principal component obtained before rotation accounted for 26.30% of the total factor loadings, which did not exceed the critical value of 40%. This indicates that there was no serious common method bias in the questionnaire survey results of this study.

### 4.2 Descriptive and correlation analyses

Table 1 lists the results of descriptive statistics in this study. The overall score for parental educational expectations was 4.42, and the mean value for physical and mental health (*M* = 4.63) was higher than that for future development (*M* = 4.34), indicating that parents are striving to find a balance and plan education more rationally. The overall score for primary school students’ social-emotional competence was 4.42, indicating that primary school students generally have good social-emotional competence. The overall score for primary school students’ peer relationships was 4.01, reflecting that primary school students are currently able to establish healthy and friendly peer interactions and have stable relationships. The score for primary school students’ academic self-efficacy was 4.03, indicating that most primary school students are confident in their own learning.

**Table 1:** Variable description analysis table (*N* = 1,653).

Variable	Dimension	*M*	SD	Skew	Kurt
Parental educational expectations	Future development	4.34	0.84	−1.46	0.60
	Physical and mental Health	4.63	0.64	−2.49	2.01
	Total value	4.42	0.53	−0.53	1.24
Peer relationships	—	4.01	0.66	−0.60	−0.17
Academic self-efficacy	—	4.03	0.74	−0.67	−0.08
Primary school students’ social–emotional competence	—	4.42	0.60	−1.33	2.16

Table 2 lists the results of the correlation analysis of the variables in this study. Parental educational expectations were significantly positively correlated with primary school students’ social-emotional competence, peer relationships, and academic self-efficacy (*r* = 0.49, *p* < 0.01; *r* = 0.30, *p* < 0.01; *r* = 0.43, *p* < 0.01). Peer relationships were significantly positively correlated with primary school students’ social-emotional competence and academic self-efficacy (*r* = 0.47, *p* < 0.01; *r* = 0.38, *p* < 0.01). Academic self-efficacy was significantly positively correlated with primary school students’ social-emotional competence (*r* = 0.71, *p* < 0.01). The correlation coefficients between the variables were all less than 0.80, and the VIF values were between 1.00 and 1.33, all less than 5. Therefore, there was no serious collinearity between the variables in this study.

**Table 2:** Correlation analysis of variables (*N* = 1,653).

Variable	1	2	3	4
1. Parental educational expectations	1			
2. Primary school students’ social-emotional competence	0.49^**^	1		
3. Peer relationships	0.30^**^	0.47^**^	1	
4. Academic self-efficacy	0.43^**^	0.71^**^	0.38^**^	1

*p* < 0.05, **p < 0.01, ***p < 0.001.

### 4.3 Analysis of chain mediation effects

#### 4.3.1 Model fit analysis

In order to effectively manage measurement errors, this study used structural equation modeling to test the chain mediation effect. Using Amos 28.0 software, 5,000 repeated samples were taken using the Bootstrap method, and a 95% confidence interval was set, and the results are shown in Table 3. The data show that *X^2^* = 306.16, *df* = 38, CFI = 0.96, AGFI = 0.94, TLI = 0.94, RMSEA = 0.07, SRMR = 0.03, NFI = 0.96, IFI = 0.96, GFI = 0.97. These indicators suggest that the model fits well, providing strong support for further analysis and enhancing the credibility and interpretability of the research conclusions.

**Table 3:** Structural equation model fit table.

Model	*X^2^*	*df*	*N*	CFI	AGFI	TLI	RMSEA	SRMR	NFI	IFI	GFI
	306.16	38	1,653	0.96	0.94	0.94	0.07	0.03	0.96	0.96	0.97

#### 4.3.2 Structural equation modeling results analysis

**Figure 2:**
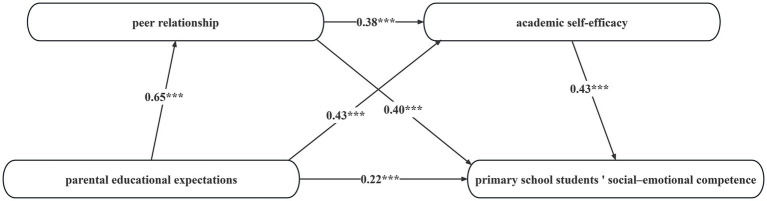
Structural equation model diagram.

After constructing the structural model and analyzing the data using AMOS 28.0, the results are shown in Figure 2. The results show that parental educational expectations have a significant positive effect on primary school students’ social-emotional competence, peer relationships, and academic self-efficacy (*β* = 0.22, *p* < 0.001; *β* = 0.65, *p* < 0.001; *β* = 0.43, *p* < 0.001), thus confirming hypothesis H1. In addition, peer relationships have a significant positive effect on primary school students’ academic self-efficacy and social-emotional competence (*β* = 0.38, *p* < 0.001; *β* = 0.40, *p* < 0.001). Compared with parental educational expectations, peer relationships have a weaker effect on primary school students’ academic self-efficacy, but a stronger direct effect on primary school students’ social-emotional competence. Academic self-efficacy has a significant positive effect on primary school students’ social-emotional competence (*β* = 0.43, *p* < 0.001).

#### 4.3.3 Analysis of mediating effects

The Bootstrap method was employed to examine the effect pathways, and the results are shown in Table 4. The direct effect of parental educational expectations on primary school students’ social-emotional competence was significant, with a Bootstrap 95% confidence interval excluding 0, an effect value of 0.22 (*p* < 0.001), accounting for 28.0% of the total effect. The total indirect effect of peer relationships and academic self-efficacy was significant, with a Bootstrap 95% confidence interval not containing 0 and an effect value of 0.56, accounting for 72.0% of the total effect. Parental educational expectations affect primary school students’ social-emotional competence through three paths: parental educational expectations → peer relationships → primary school students’ social-emotional competence, with an effect value of 0.26 (*p* < 0.01), accounting for 34% of the total effect. Therefore, hypothesis H2 was supported. Parental educational expectations → academic self-efficacy → primary school students’ social-emotional competence, with an effect value of 0.19 (*p* < 0.001), accounting for 24.1% of the total effect, therefore, hypothesis H3 was confirmed. Parental educational expectations → peer relationships → academic self-efficacy → primary school students’ social-emotional competence, with an effect value of 0.11 (*p* < 0.01), accounting for 13.9% of the total effect, therefore, hypothesis H4 was validated.

**Table 4:** Mediation effect path analysis.

Type of effect			95% Confidence interval		
Path	*β*	SE	LL	UL	Path effect proportion
Parental educational expectations → Primary school students’ social-emotional competence	0.22^***^	0.10	0.09	0.44	28.0%
Parental educational expectations → Peer relationships → Primary school students’ social-emotional competence	0.26^**^	0.05	0.05	0.09	34.0%
Parental educational expectations → Academic self-efficacy → Primary school students’ social-emotional competence	0.19^***^	0.05	0.18	0.25	24.1%
Parental educational expectations → Peer relationships → Academic self-efficacy → Primary school students’ social-emotional competence	0.11^**^	0.03	0.04	0.07	13.9%

*p* < 0.05, **p < 0.01, ***p < 0.001.

## 5 Discussion

### 5.1 Parental educational expectations shift from academic performance to physical and mental health

This study found that the mean scores for the physical and mental health dimensions of parental educational expectations were higher than those for the future development dimension, indicating that current parental expectations may place greater emphasis on students’ physical and mental health. This trend aligns with the direction of current educational policies. The “Double Reduction” policy, through strict regulations, has reduced students’ academic burdens and fundamentally altered the exam-oriented education mindset at the institutional level. Simultaneously, the new curriculum standards explicitly emphasize the “five-fold development” approach, establishing moral, intellectual, physical, aesthetic, and labor education as statutory goals. Reforms to the evaluation system further guide parents in adjusting their parental educational expectations. In recent years, mental health issues among adolescents have become increasingly prominent. The China National Mental Health Development Report (2022) reveals rising rates of depression and suicidal ideation among primary school students (Fu et al., 2023). This grim reality has accelerated policy internalization, prompting parents to rethink the risks of “test-score obsession” and gradually recognize the critical role of non-cognitive skills—such as social-emotional competence—in students’ long-term development and social adaptability. Society’s trend toward diversifying talent definitions and critical discussions about the “adult baby” phenomenon resonate with policy directions, collectively reinforcing parents’ emphasis on physical and mental health dimensions within parental educational expectations.

### 5.2 The dominant role of peer relationships in the development of social-emotional competence among primary school students

Peer relationships contributed 34% of the mediating effect in the path from parental educational expectations to primary school students’ social-emotional competence, which was significantly higher than the direct effect (28%) and other indirect paths, highlighting the importance of peer relationships. Parent–child interactions are essentially vertical relationships that are instructive and restrictive, with primary school students often taking a passive and receptive role in these interactions. In contrast, peer relationships are horizontal relationships that are egalitarian and consultative (Feinberg et al., 2019). By observing and imitating their peers’ behaviors in dealing with emotions, children are more likely to develop internal identification and motivation to actively practice, thereby effectively improving their social-emotional competence. In addition, China’s education orientation of “cultivating people with virtue” and the guidance of peer relationships in courses such as “Morality and the Rule of Law” may further amplify the value of peer relationships. Therefore, the role of parents may need to shift from the traditional “direct teacher” to a “supportive environment shaper,” indirectly promoting the development of students’ social-emotional competence by actively creating a healthy peer interaction environment.

### 5.3 The chain-mediated role of peer relationships, academic self-efficacy, and parental educational expectations on primary students’ social-emotional competence

The chain mediation effect of peer relationships and academic self-efficacy between parental educational expectations and primary school students’ social-emotional competence is partially supported by the findings of Huang et al. (2021) and Zhou et al. (2023). Positive peer relationships enhance academic self-efficacy by providing social reference and emotional support (Marsh et al., 2011), which in turn translates into more adaptive learning strategies and emotional regulation abilities, ultimately nurturing the development of social-emotional competence. Research also shows that parental educational expectations can influence students’ positioning in peer relationships (Yang and Zhu, 2013), However, it is crucial to acknowledge its potential as a “double-edged sword”: an excessive focus on academic achievement may induce competitive rather than supportive peer relationships, and poor peer relationships may lead to a decrease in adolescents’ academic self-efficacy, affecting their academic performance (Llorca et al., 2017). If students fail to meet their parents’ expectations, their academic self-efficacy may also be directly weakened.

## 6 Conclusions and recommendations

### 6.1 Conclusion

This study uses the ecosystem theory as its theoretical framework. A survey of third- to fifth-grade primary school students in Zhejiang Province, China, shows that: First, in China, parental educational expectations are shifting from academic performance to comprehensive quality, which promotes the development of social-emotional competence through direct and triple indirect pathways. Second, compared with parental educational expectations, peer relationships have a weaker influence on primary school students’ academic self-efficacy, but a stronger influence on primary school students’ social-emotional competence. Third, the mediating effect of peer relationships accounts for 34%, indicating that parental educational expectations significantly improve the quality of primary school students’ peer relationships, thereby enhancing their social-emotional competence. Fourth, the chain mediation effect of peer relationships and academic self-efficacy accounts for 13.9%, indicating that parental educational expectations also indirectly influence primary school students’ social-emotional competence through this path.

### 6.2 Recommendations

#### 6.2.1 Family-school-community collaboration in building supportive environments to Foster students’ social-emotional competence

To effectively guide parental educational expectations and support students’ social-emotional competence, policymakers and educational institutions should jointly establish a collaborative support environment linking families, schools, and communities. Schools should proactively maintain regular communication with communities and families—for instance, by hosting periodic “Family-School-Community Collaboration Forums” and distributing family emotional education guides—to align parental educational expectations with emotional education principles. Communities should actively build child-friendly environments by establishing more public activity spaces and peer support groups. This encourages students to practice key social-emotional skills—such as collective decision-making and collaborative problem-solving—in authentic social settings. Parents participating in these community activities can gradually shift their educational perspectives, moving from a narrow focus on academic performance to providing comprehensive support for their children’s holistic growth.

#### 6.2.2 Redesign the classroom social ecosystem to enhance the positive influence of peer relationships

Given the characteristics of China’s classroom-based instruction system, there is an urgent need to develop strategies for optimizing classroom social ecosystems to enhance the role of peer relationships in fostering primary school students’ social-emotional competencies. Education authorities should vigorously support teacher training to improve educators’ social-emotional teaching capabilities, integrate peer relationship building into daily instruction, and develop localized campus relationship monitoring systems. These systems should conduct regular assessments of classroom social atmospheres and implement targeted interventions for students experiencing interpersonal tensions. Second, schools should establish scientific mechanisms for dynamic peer relationship monitoring. Utilizing social network analysis to identify marginalized students, they should promote positive interactions through peer mentoring programs. Institutionalizing class integration activities will deepen peer bonds through experiential learning. Homeroom teachers should actively shift classroom management from competition to cooperation, implementing team incentive mechanisms and honor systems to strengthen collective identity and mutual support behaviors among students.

#### 6.2.3 Establish a dynamic promotion mechanism for peer relationships and academic self-efficacy to achieve a virtuous cycle of social-emotional competence development

Parental educational expectations should be supportive and developmentally oriented, leveraging peer groups to build learning communities that both advance academic progress and foster social skills. Given the characteristics of classroom instruction, parents can gradually cultivate leadership abilities within student peer groups: from small-group collaboration to class management, enhancing responsibility awareness while providing strategic guidance. When students encounter academic setbacks, design peer-support intervention plans. Through role modeling and experience sharing, help students reconstruct their cognitive frameworks, transforming setbacks into growth momentum. Parents should embrace developmental assessment principles, guiding students to establish phased goal systems. This allows students to strengthen their academic self-efficacy through incremental breakthroughs. Additionally, utilize attribution training to help them develop positive cognitive patterns.

### 6.3 Theoretical contributions

The main contributions of this study are reflected in three aspects. First, this study reveals the positive predictive effect of parental educational expectations on social-emotional competence. Based on the ecosystem theory, this study constructs and verifies a theoretical model (see Figure 2), which not only enriches theoretical research in the field of family education but also provides guidance for educational practice. Second, this study innovatively reveals the chain mediation effect of “parental educational expectations-peer relationships-academic self-efficacy-social-emotional competence,” which breaks through the limitations of single mediation and reveals the internal mechanism of multiple factors synergistically influencing social-emotional competence, providing a new perspective for understanding the psychological development of primary school students. In addition, this study focuses on the social-emotional competence of primary school students, filling the gap in previous research on factors affecting its development, especially from the perspective of parental educational expectations, and providing new ideas for educational practice.

### 6.4 Limitations and implications

First, due to limited resources, this study only selected some students in Zhejiang Province, China as research subjects, which may affect the generality and generalizability of the research results. Future studies can expand the sample range and conduct cross-regional and cross-cultural comparative studies to verify and enrich the research findings. Finally, future studies can be extended to kindergarten and middle school levels to systematically explore the impact of parental educational expectations on the social-emotional competence of students at different educational stages, providing more targeted guidance for educational practice.

## Data Availability

The raw data supporting the conclusions of this article will be made available by the authors without undue reservation.
