# Correction: The influence of parental educational expectations on primary school students' social-emotional competence: the chain mediation effect of peer relationships and academic self-efficacy

**DOI:** 10.3389/fpsyg.2025.1731844

**Published:** 2025-11-18

**Authors:** Lianghong Yang, Kaizhou Chen, Suling Ma, Yan Lei

**Affiliations:** Faculty of Teacher Education, Lishui University, Lishui, China

**Keywords:** primary school students, parental educational expectations, social-emotional competence, peer relationships, academic self-efficacy, chain mediation effect

There was a mistake in Table 1 as published. The apostrophe in the variable name “Primary school students' social-emotional competence” was incorrectly formatted. The corrected Table 1 appears below.

**Table 1:** Variable description analysis table (*N* = 1,653).

**Variable**	**Dimension**	***M***	**SD**	**Skew**	**Kurt**
Parental educational expectations	Future development	4.34	0.84	−1.46	0.60
	Physical and mental health	4.63	0.64	−2.49	2.01
	Total value	4.42	0.53	−0.53	1.24
Peer relationships	—	4.01	0.66	−0.60	−0.17
Academic self-efficacy	—	4.03	0.74	−0.67	−0.08
Primary school students' social–emotional competence	—	4.42	0.60	−1.33	2.16

In Section 3.1 Participants, the percentage of third-grade students was incorrectly reported as 31.1%, and an extraneous sentence that requires deletion was included at the end of the section: “After verification, there are 515 students in Grade 3, accounting for 31.2%”.

A correction has been made to the section 3.1 Participants, last paragraph:

“Among these, 50.8% were male and 49.2% were female; third-grade students accounted for 31.2%, fourth-grade students for 41.3%, and fifth-grade students for 27.5%.”

The original version of this article has been updated.

